# Pressure‐Induced Superconductivity and Topological Quantum Phase Transitions in the Topological Semimetal ZrTe_2_

**DOI:** 10.1002/advs.202301332

**Published:** 2023-11-09

**Authors:** Shihao Zhu, Juefei Wu, Peng Zhu, Cuiying Pei, Qi Wang, Donghan Jia, Xinyu Wang, Yi Zhao, Lingling Gao, Changhua Li, Weizheng Cao, Mingxin Zhang, Lili Zhang, Mingtao Li, Huiyang Gou, Wenge Yang, Jian Sun, Yulin Chen, Zhiwei Wang, Yugui Yao, Yanpeng Qi

**Affiliations:** ^1^ School of Physical Science and Technology ShanghaiTech University Shanghai 201210 China; ^2^ Centre for Quantum Physics Key Laboratory of Advanced Optoelectronic Quantum Architecture and Measurement (MOE) School of Physics Beijing Institute of Technology Beijing 100081 China; ^3^ Beijing Key Lab of Nanophotonics and Ultrafine Optoelectronic Systems Beijing Institute of Technology Beijing 100081 China; ^4^ Material Science Center Yangtze Delta Region Academy of Beijing Institute of Technology Jiaxing 314011 China; ^5^ ShanghaiTech Laboratory for Topological Physics ShanghaiTech University Shanghai 201210 China; ^6^ Center for High Pressure Science and Technology Advanced Research Shanghai 201203 China; ^7^ Shanghai Synchrotron Radiation Facility Shanghai Advanced Research Institute Chinese Academy of Sciences Shanghai 201203 China; ^8^ National Laboratory of Solid State Microstructures School of Physics and Collaborative Innovation Center of Advanced Microstructures Nanjing University Nanjing 210093 China; ^9^ Department of Physics Clarendon Laboratory University of Oxford Parks Road Oxford OX1 3PU UK; ^10^ Shanghai Key Laboratory of High‐resolution Electron Microscopy ShanghaiTech University Shanghai 201210 China

**Keywords:** high pressure, superconductivity, topological materials, transition metal dichalcogenides

## Abstract

Topological transition metal dichalcogenides (TMDCs) have attracted much attention due to their potential applications in spintronics and quantum computations. In this work, the structural and electronic properties of topological TMDCs candidate ZrTe_2_ are systematically investigated under high pressure. A pressure‐induced Lifshitz transition is evidenced by the change of charge carrier type as well as the Fermi surface. Superconductivity is observed at around 8.3 GPa without structural phase transition. A typical dome‐shape phase diagram is obtained with the maximum *T*_c_ of 5.6 K for ZrTe_2_. Furthermore, the theoretical calculations suggest the presence of multiple pressure‐induced topological quantum phase transitions, which coexists with emergence of superconductivity. The results demonstrate that ZrTe_2_ with nontrivial topology of electronic states displays new ground states upon compression.

## Graphical abstract

A pressure‐induced Lifshitz transition and superconductivity is observed in ZrTe_2_ under high pressure without structural phase transition. The theoretical calculations suggest the presence of multiple pressure‐induced topological quantum phase transitions, which coexists with emergence of superconductivity. The results demonstrate that ZrTe_2_ with nontrivial topology of electronic states display new ground states upon compression and have potential applications in next‐generation devices.

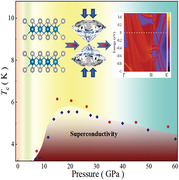

## 1 Introduction

In the past decades, the layered transition metal dichalcogenides (TMDCs: *M*X_2_, *M* = Mo, W, Ta, Zr, Hf, etc., and X = S, Se, or Te) have attracted tremendous attention owing to their rich physics and potential device applications.^[^ ^1^, ^2^, ^3^, ^4^, ^5^, ^6^, ^7^, ^8^ ^]^ The diversity of electronic properties of TMDCs includes the charge density wave (CDW),^[^ ^9^, ^10^, ^11^ ^]^ the magnetism,^[^ ^12^, ^13^, ^14^ ^]^ and the superconductivity (SC).^[^ ^15^, ^16^ ^]^ Recent studies have shown that TMDCs exhibit nontrivial topology,^[^ ^17^, ^18^, ^19^, ^20^ ^]^ making the study of these materials even more intriguing. In particular, superconductivity was observed successfully in TMDCs, either in stoichiometric compounds at ambient or under high pressure, or by doping/intercalation individual layers.^[^ ^21^, ^22^, ^23^, ^24^ ^]^ Therefore, TMDC family provides an exotic platform to study the relation between topologically non‐trivial state and superconductivity and even exploration of topological superconductivity (TSC).^[^ ^25^, ^26^, ^27^, ^28^ ^]^

Among TMDCs, ZrTe_2_ has been relatively little investigated; however, it is predicted to possess non‐trivial band topology recently. Although theoretical calculations indicated that ZrTe_2_ is a topological crystalline insulator protected by crystalline symmetry,^[^ ^29^, ^30^, ^31^ ^]^ however, angle‐resolved photoemission spectroscopy (ARPES) studies have revealed that ZrTe_2_ is a topological semimetal with approximately equal electron and hole carrier densities.^[^ ^32^, ^33^ ^]^ Tian et al. performed nuclear magnetic resonance (NMR) experiments and supported ZrTe_2_ as a quasi‐2D Dirac semimetal with a nodal line between *Г* and *A*.^[^ ^34^ ^]^ Negative magnetoresistivity has been observed in both thin films, single crystals and nanoplates prepared by mechanical exfoliation,^[^ ^35^, ^36^, ^37^ ^]^ further indicating its topological semimetal features. More interestingly, bulk superconductivity was observed by intercalating Cu or Ni in the van der Waals gap, indicating a possible candidate for TSC.^[^ ^22^, ^38^ ^]^

The application of pressure can effectively tune the crystal structures and the corresponding electronic states in a valid and systematic fashion, and its related studies on *M*X_2_, indeed, have given rise to many novel physical phenomena.^[^ ^23^, ^39^, ^40^, ^41^, ^42^, ^43^, ^44^ ^]^ To date, the high‐pressure properties of ZrTe_2_ have not been well explored. Here, we systematically explore the structure and electronic properties of topological TMDC ZrTe_2_ single crystal under high pressure. Room‐temperature synchrotron x‐ray diffraction and Raman scattering measurements reveal the stability of the hexagonal CdI_2_‐type structure up to 49.3 GPa. We demonstrate a pressure‐induced Lifshitz transition revealed by the sign change of the charge carrier type and the Fermi surface. A superconducting transition is observed in ZrTe_2_ at around 8.3 GPa and *T*_c_ reaches the maximum of 5.6 K around 19.4 GPa. Through the first‐principles calculations, we find that the application of pressure alters the electronic properties and leads to multiple topological quantum phase transitions in ZrTe_2_.

## 2 Results and Discussion

**Figure 1:**
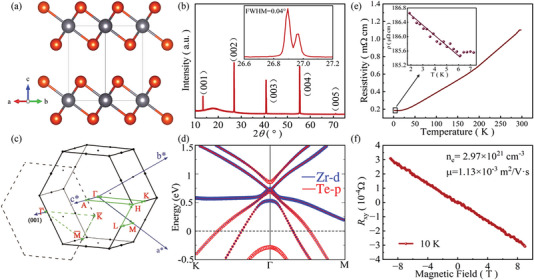
a) The crystal structure of ZrTe_2_ (Zr: gray; Te: orange). b) X‐ray diffraction peaks from the ab plane of ZrTe_2_ single crystal. The inset shows the details of the (002) reflection. c) The bulk Brillouin zone and its projections onto the conventional cell (001) surface. d) Calculated band structure of ZrTe_2_ with spin‐orbit coupling (SOC). e) Temperature dependence of resistivity for ZrTe_2_. Inset: The evolution of the resistivity at low temperature. f) Transversal Hall resistance of ZrTe_2_ single crystal at 10 K.

At ambient pressure, ZrTe_2_ adopts a hexagonal CdI_2_‐type structure with space group *P‐*3*m*1 (No. 164) as shown in **Figure** **1**a. The synthesized ZrTe_2_ sample is characterized by the XRD experiments. The XRD pattern of ZrTe_2_ single crystal is shown in Figure 1b. The (00l) plane is a natural cleavage facet of as‐grown single crystals. The full width at half maximum (FWHM) of (002) peak is only 0.04◦ (inset of Figure 1b), indicating the high quality of our samples. The *c*‐axis lattice constant is 6.625 ± 0.005 Å, consistent with the previous reports.^[^ ^37^ ^]^ The energy dispersive x‐ray spectrometry (EDXS) data in Figure S1 (Supporting Information) gives the ratio of Zr:Te as 1:2.01. Figure 1d presents the band structure of ZrTe_2_ calculated along high‐symmetry lines in the first Brillouin zone (BZ, Figure 1c). We can observe a band inversion around the *Γ* point, confirming topological semimetal behaviors. Figure 1e exhibits the temperature dependence of resistivity for ZrTe_2_ crystal. A metallic behavior is observed with decreasing temperature followed by the resistive upturn below ≈6 K. The resistivity behavior shown here is in line with the previously reported data, which may derive from weak Kondo effect.^[^ ^37^ ^]^ We further conduct the transversal Hall resistance at 10 K (Figure 1f) and the ZrTe_2_ is dominated by electron‐type carriers with the electron concentration *n*_e_ ≈ 2.97 × 10^21^ cm^−3^ at 10 K.

**Figure 2:**
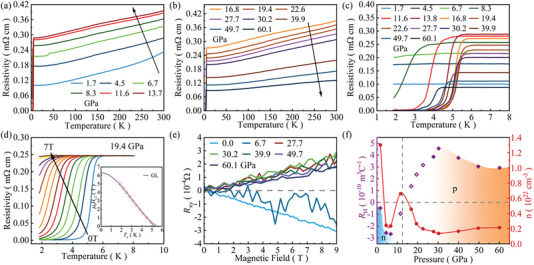
Electrical resistivity of ZrTe_2_ as a function of temperature a) below and b) above 13.7 GPa. c) Temperature‐dependent resistivity of ZrTe_2_ in the vicinity of superconductivity. d) Temperature dependence of resistivity under different magnetic fields for ZrTe_2_ at 19.4 GPa. The inset shows the results of Ginzburg–Landau fitting. e) Hall resistance of ZrTe_2_ as a function of magnetic field under selected pressures at 10 K. f) Pressure dependence of Hall coefficient and carrier concentration at 10 K for ZrTe_2_. Hollow points represent noise effects.

Under high pressure, we measure the temperature dependence of resistivity *ρ*(T) for ZrTe_2_ crystals. As depicted in **Figure** **2**a,b, ZrTe_2_ keeps metallic behavior up to 60.1 GPa, while the normal state of resistivity exhibits a non‐monotonic evolution with increasing pressure. Increasing the pressure initially induces continuous enhancement of the overall magnitude of *ρ* with a maximum occurring at 13.7 GPa. Upon further increasing the pressure, the resistivity starts to decrease gradually. As pressure increases up to 8.3 GPa, a sharp drop of resistivity in ZrTe_2_ is observed at the lowest temperature (experimental *T*_min_ = 1.8 K), indicating the emergence of superconductivity, and zero resistivity is obtained when the pressure enhances to 11.6 GPa. The critical temperature *T*_c_ reaches the maximum *T*_c_ of 5.6 K around 19.4 GPa and then decreases with pressure, as plotted in Figure 2c (*T*_c_ is referred to *T*_c_^onset^ defined as the temperature at 90% of the residual resistivity in this paper). The measurements on different samples of ZrTe_2_ from two independent runs provide reproducible and consistent results, confirming the superconductivity transition under pressure (Figure S2, Supporting Information). To gain insights into the superconducting transition, we applied the magnetic field for ZrTe_2_ subjected to 19.4 GPa. As shown in Figure 2d, *T*_c_ is gradually suppressed with the enhancement of magnetic fields and the superconductivity extinguishes under the magnetic field *µ*_0_ *H* = 7 T. We tried to use the Ginzburge–Landau formula to fit the data (inset of Figure 2d). The estimation of *µ*_0_ *H*_c2_ at 0 K is ≈ 6.1 T, and the Ginzburg–Landau coherence length *ξ*_GL_(0) is 7.3 nm. High‐pressure Hall resistivity measurements were further carried out to extract the evolution of charge carriers in the pressurized ZrTe_2_. Figure 2e and Figure S3 (Supporting Information) show the Hall resistivity curves *R*_xy_(H) measured at 10 K under various pressures. At low pressure region, the *R*_xy_(H) curve exhibits a negative slope, indicating an electron‐dominated feature of the electrical transport. This is in agreement with the carriers type at ambient pressure. When the pressure is above 27.7 GPa, the Hall resistance slope becomes positive, suggesting the dominance of hole‐type carriers. In the first run of Hall measurements, *R*_xy_ is affected by the noise between 6.7 and 27.7 GPa due to the competition between two types of carriers caused by pressure gradient. We repeated the high‐pressure Hall measurements with sodium chloride as pressure transmitting medium. Compared to previous Hall measurements, we reduced the noise influence, as shown in Figure S3b (Supporting Information). The slope of *R*_xy_ changes from negative to positive at 11.2 GPa, and the sign change of the *R*_xy_(H) demonstrates the change of charge carrier type, which indicates the change of the Fermi surface topology. This variation could be viewed as a signature of the Lifshitz transition.^[^ ^41^, ^45^ ^]^ Pressure dependence of Hall coefficient and carrier concentration at 10 K are summarized in Figure 2f.

**Figure 3:**
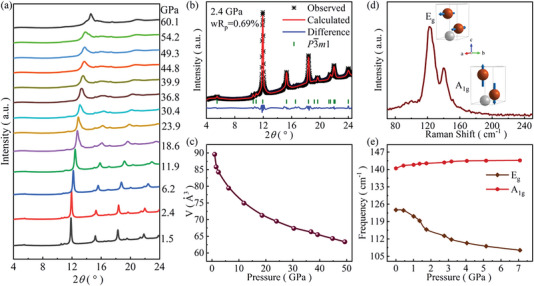
a) High‐pressure XRD patterns of ZrTe_2_ up to 60.1 GPa at room temperature. The X‐ray diffraction wavelength *λ* is 0.6199 Å. b) Rietveld refinement of XRD pattern at 2.4 GPa. The red solid line and black stars represent the calculated and experimental data, respectively, and the blue solid lines are the residual intensities. The vertical bars are the diffraction peak positions. c) Pressure dependence of unit‐cell volume. d) Raman spectra of ZrTe_2_ at ambient pressure (Zr: gray; Te: orange). e) Pressure dependence of vibration modes frequencies of ZrTe_2_.

To examine the thermodynamic stability of the ZrTe_2_ phase and whether the pressure‐induced SC is associated with structural phase transition, we performed in situ high‐pressure powder XRD measurements at room temperature. **Figure** **3**a displays the high‐pressure synchrotron XRD patterns of ZrTe_2_ up to 60.1 GPa. A representative refinement at 2.4 GPa is presented in Figure 3b. All the diffraction peaks can be indexed well to ambient structure (space group *P‐*3*m*1, No. 164) based on Rietveld refinement with General Structure Analysis System (GSAS) software package. All the XRD peaks continuously shift toward higher angles without new peaks appearing when the pressure increases up to 49.3 GPa, indicating the absence of structural phase transition in the pressurized ZrTe_2_. Above 49.3 GPa, the signal intensity of the main peak deviates from the symmetry of *P*‐3*m*1. We expect to study this structural transitions in the future. Figure 3c shows the pressure (*P*) dependence of volume (*V*). Upon compression from 1.5 to 49.3 GPa, the overall volume decreases by 29% without volume collapse. In addition, We have performed single‐crystal XRD under 5.8 and 14.3 GPa (Figure S14 and Table S1, Supporting Information). The results of single crystal XRD demonstrate that ZrTe_2_ retains *P*‐3*m*1 up to 14.3 GPa. The stability of ZrTe_2_ was also confirmed by in situ Raman spectroscopy measurements. As shown in Figure 3d, the Raman spectra at ambient pressure contain two characteristic peaks, which are due to the in‐plane mode *E*_g_ and the out‐of‐plane mode *A*_1g_ of the ZrTe_2_ structure; this is also in agreement with a previous report.^[^ ^46^ ^]^ The frequencies of both vibrational modes move gradually without discontinuities as pressure increases (Figure S4, Supporting Information) indicating the robustness of structure in the whole studied pressure range at room temperature. Interestingly, *E*_g_ mode displays the opposite trend and shows redshift behavior when the pressure is raised. This pressure‐induced phonon softening is probably associated with emergence of superconductivity in the pressurized ZrTe_2_.

**Figure 4:**
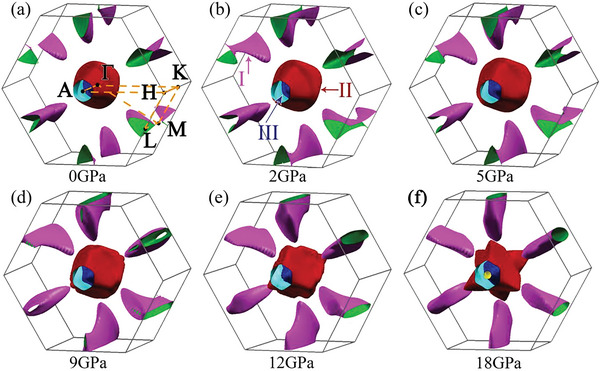
The Fermi surface of ZrTe_2_ at various pressures. The Brillouin zone path is shown in (a), and different Fermi surfaces are indexed as I, II, and III in (b), respectively.

To further understand the transport behavior of ZrTe_2_ under high pressure, we first conducted the Fermi surface calculations at various pressures (**Figure** **4**). The Fermi surfaces are indexed as I, II, and III in Figure 4b. The decomposed Fermi surfaces are plotted in Figures S5–S8 (Supporting Information), and the Fermi surface IV is enclosed by the Fermi surface III. The Fermi surface I forms a connection around 2 GPa (Figure 4b). The pocket enlarges with the pressure and closes around 12 GPa (Figure 4e). The Fermi surface II transforms from shuttle shape to David‐star shape at 18 GPa (Figure S6, Supporting Information). The hexagon (Figure S7c, Supporting Information) in Fermi surface III becomes a David‐star at 12 GPa (Figure S7e, Supporting Information), and an opening emerges at *A* point under 18 GPa (Figure S7f, Supporting Information). The Fermi surface IV emerges around 5 GPa (Figure S5c, Supporting Information) and vanishes about 12 GPa (Figure S5e, Supporting Information). The evolution of the Fermi surface under high pressure could be the signature of the Lifshitz transitions, and the reshape of Fermi surface I, III, and IV at 12 GPa is in line with the normal state anomalies in our resistivity measurements.

**Figure 5:**
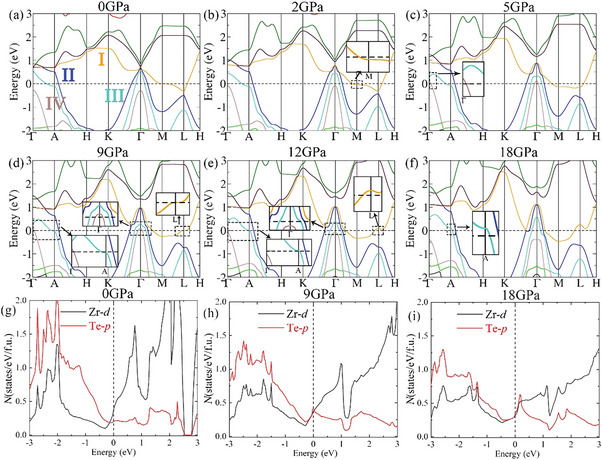
a–f) The band structures of ZrTe_2_ at various pressures. The orange, blue, cyan, and brown bands are indexed as band I, II, III, and IV, respectively. The insets show the details of the band structures. g–i) The partial density of states (PDOS) at various pressures. The black and red solid lines are the PDOS of the *d* electrons of Zr atoms and the *p* electrons of Te atoms, respectively.

Next, we calculated the band structures of ZrTe_2_ at various pressures and the details are shown in **Figure** **5**a–f. The band indexes I, II, III, and IV are in line with the Fermi surfaces. The band I crosses the Fermi energy along *Γ*‐*M* at 2 GPa (Figure 5b), causing the connection in Figure 4b, and the pocket enclosing at *L* point (Figure 4e) can be identified to a *p*‐type conversion of band I (Figure 2e,f). As for the David‐star in the Fermi surface II, the band structures on the *k*_z_ = 0.25 plane (Figure S6g–i, Supporting Information) indicate a saddle point around the Fermi energy under 18 GPa (Figure S6i, Supporting Information). The reshape of the Fermi surface III (Figure S7, Supporting Information) is owing to the saddle point along *Γ*‐*M* (Figure 5e), and the opening at 18 GPa is due to the *p*‐type crossing at the Fermi energy around *A* point (Figure 5e,f). The emerging and vanishing of Fermi surface IV originates from the variation of band IV around *Γ* point from 5 to 12 GPa (Figure 5c–e). Thus, band structures calculation provides details for the Lifshitz transitions of Fermi surface topology, and the *p*‐type conversion at *A* (Figure S10, Supporting Information) and *L* (Figure S9, Supporting Information) points is in line with the charge carrier type transition under high pressure.

Moreover, we calculated the orbital contribution around *A*, *Γ*, and *L* points (Figures S9–S11, Supporting Information). Around the Fermi energy, the main distribution is from the *p* electrons of Te atoms, such as the band I at *L* point (Figure S9, Supporting Information), band III at *A* point (Figure S10, Supporting Information) and band IV at Γ point (Figure S11, Supporting Information). Accordingly, we calculated the partial density of states (PDOS) at various pressures (Figure 5g,h). The PDOS is more diverged under high pressure, and a peak emerges on the Fermi energy around 9 GPa. This peak is from the Te*‐p* electrons and could contribute to the pressure‐induced superconductivity. Besides, we calculated the charge density between the inter‐layer and intra‐layer Te atoms under high pressure (Figure S12, Supporting Information). Compared with intra‐layer Te atoms, more electrons are distributed between inter‐layer Te atoms. This is empirically true since the inter‐layer distance is easier to compress in layered TMDCs. Such novel bonds explain the DOS peak of Te‐*p* electrons around the Fermi energy, which could be favorable for the superconducting transition through electron‐phonon coupling. Hence, our results demonstrated that the anisotropic compression behaviors in ZrTe_2_ cause the redistribution of Te‐*p* electrons. It leads to the reshape of the band structures and the Fermi surface topology, which is in agreement with the transport anomalies of normal state and the carriers type conversion under high pressure. The anisotropic compression causes the bonding and PDOS elevation around Fermi energy of Te‐*p* electrons as well, which is in consistent with the pressure‐induced superconductivity in our experiments.

**Figure 6:**
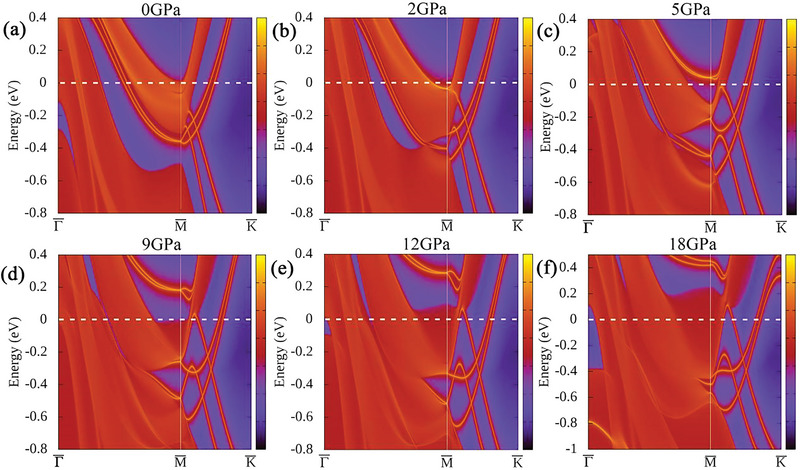
The surface states of ZrTe_2_ on the (001) plane around $\bar{M}$ point at various pressures.

Meanwhile, we observed a gap opening at *Γ* point (Figure S11, Supporting Information) and the band inversion at *L* point (Figure S9, Supporting Information), suggesting potential topological properties. We calculated the $Z_{2}$ invariant of band I and II up to 50 GPa. The detailed results are shown in **Table** **1**. The band I keeps topologically trivial within the pressure range. For band II, it transforms from topologically non‐trivial state at 30 GPa to trivial state at 50 GPa, and the variation of $Z_{2}$ invariant around 2 GPa (1→0→1) suggests the topological states transition. This is similar to the results observed in *β*‐Bi_4_I_4_.^[^ ^47^ ^]^ The surface states on (001) plane at various pressures are shown in **Figure** **6** and Figure S13 (Supporting Information). We could observe the split and cross of surface states around $\bar{M}$ point with the pressure increasing, while, the surface states around the $\bar{\Gamma }$ point are more complex as shown in Figure S9 (Supporting Information). Therefore, the topological properties of ZrTe_2_ could be modulated by high pressure. More importantly, our results shown here demonstrate the coexistence of non‐trivial topology and superconductivity in ZrTe_2_ upon compression. Our study will stimulate further studies, such as the quantum oscillations^[^ ^48^, ^49^ ^]^ and Josephson effect^[^ ^50^, ^51^ ^]^ under high pressure, to explore potential topological superconductivity and Majorana fermions.

**Table 1:** The $Z_{2}$ invariant of band II under different pressures.

Pressure [GPa]	Time reversal invariant planes						$Z_{2}$ index (υ_0_;υ_1_υ_2_υ_3_)
	*k*_x_ = 0.0	*k*_y_ = 0.5	*k*_y_ = 0.0	*k*_y_ = 0.5	*k*_z_ = 0.0	*k*_z_ = 0.5	
0	0.0	0.0	0.0	0.0	1.0	0.0	(1;000)
2	1.0	0.0	1.0	0.0	1.0	1.0	(0;001)
5	0.0	0.0	0.0	0.0	0.0	1.0	(1;001)
7	1.0	0.0	1.0	0.0	0.0	1.0	(1;001)
9	1.0	0.0	1.0	0.0	0.0	1.0	(1;001)
12	1.0	0.0	1.0	0.0	0.0	1.0	(1;001)
18	0.0	0.0	0.0	0.0	0.0	1.0	(1;001)
24	0.0	0.0	0.0	0.0	0.0	1.0	(1;001)
30	0.0	0.0	0.0	0.0	0.0	1.0	(1;001)
50	0.0	0.0	0.0	0.0	0.0	0.0	(0;000)

**Figure 7:**
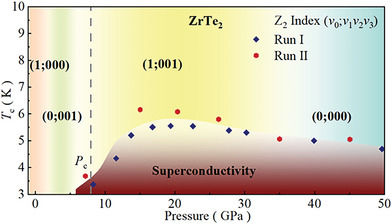
Phase diagram of ZrTe_2_. Superconducting transition emerges at around 8.3 GPa and maintains up to 60 GPa. Topological state transitions are shown as the variation of $Z_{2}$ index. A topological states transition arises around 2 GPa and topologically non‐trivial state coexists with superconducting state up to 30 GPa.

Based on the above resistivity, XRD, and theoretical calculation, the *T*‐*P* phase diagram is summarized in **Figure** **7**. These results demonstrate that high pressure dramatically alters both topological and transport properties of ZrTe_2_. Its crystal sustains a hexagonal CdI_2_‐type structure under high pressures up to 49.3 GPa, while applied pressure induces multiple topological quantum phase transitions in ZrTe_2_. More interestingly, superconductivity emerges around 8.3 GPa and *T*_c_ reaches maximum of 5.6 K around 19.4 GPa, showing a typical dome‐like evolution. The combined theoretical calculations and in situ high‐pressure measurements demonstrate the topologically non‐trivial state is accompanied by the appearance of superconductivity, making ZrTe_2_ possible platform to study topological superconductivity.

## 3 Conclusion

In summary, we discovered pressure‐induced superconductivity in topological TMDC ZrTe_2_ by combining experimental and theoretical investigations. High pressure dramatically alters the electronic state, and a pressure‐induced Lifshitz transition is evidenced by the change of charge carrier type as well as the Fermi surface. Superconductivity is observed in ZrTe_2_ at large pressure region with a dome‐shape evolution. Theoretical calculations indicated that ZrTe_2_ experiences multiple pressure‐induced topological quantum phase transitions, which coexists with superconductivity. Our results demonstrate that ZrTe_2_ with a nontrivial topology of electronic states display new ground states upon compression and have potential applications in next‐generation spintronic devices.

## 4 Experimental Section

### Crystal Growth and Sample Characterization

High‐quality single crystals of ZrTe_2_ were grown by using chemical vapor transport method. High‐purity starting materials (total amount of 0.5 g) of Zr powder and Te powder were loaded in a quartz tube with the ratio of Zr:Te = 1:2, and I_2_ with the concentration of 4 mg mL^−1^ was added as a transport agent. The tube was sealed after it was evacuated to a vacuum of 2 × 10^−4^ Pa, which was then put into a two‐zone tube furnace. The temperatures were set to be 1173 and 1073 K for the hot‐side (source) and cold‐side (crystal), respectively. After a week, the furnace was cooled to room temperature with the power supply switched off. In order to obtain crystals with high quality, surface cleaning was performed for all starting materials to remove the oxide layers formed in air.^[^ ^52^, ^53^ ^]^ The crystalline phase of ZrTe_2_ was checked by the single‐crystalline x‐ray diffraction (XRD, Cu *K*_α_, λ = 1.54184 Å). The chemical composition value of ZrTe_2_ was given by energy‐dispersive x‐ray spectra (EDX).

### High Pressure Electrical Transport Measurements

The electrical transport measurements were carried out by the van der Pauw four‐probe method using a nonmagnetic Be‐Cu alloy diamond‐anvil cell (DAC) with 200 µm culets.^[^ ^54^, ^55^, ^56^ ^]^ Thickness of the pre‐indented Be‐Cu gasket was 30 µm and a hole with diameter of about 140 µm was drilled using a pulse laser. The mixture of cubic boron nitride (*c*‐BN) powder and epoxy was loaded into the hole, and then compressed to insulate the platinum electrodes with the Be‐Cu gasket. A plate‐like single crystal was loaded into the sample chamber. Pressure was calibrated by the ruby luminescence method.^[^ ^57^ ^]^ Electrical transport measurements were performed in a commercial Physical Property Measurement System (PPMS, Quantum Design Inc.).

### High‐Pressure Structure Measurements

In situ high‐pressure x‐ray diffraction (XRD) experiments were performed on ZrTe_2_ powder sample at the BL15U1 beamline of Shanghai Synchrotron Radiation Facility (wavelength λ = 0.6199 Å). A symmetrical DAC with 200 µm culets was used with rhenium gasket. The XRD images were integrated and analyzed using the FIT2D software.^[^ ^58^ ^]^ The diffraction patterns were refined by the General Structure Analysis System (GSAS) and the graphical user interface EXPGUI.^[^ ^59^ ^]^ High‐pressure in situ Raman spectroscopy investigation on ZrTe_2_ was carried out on a Raman spectrometer (Renishaw in Via, U.K.) with a laser excitation wavelength of 532 nm as well as a low‐wavenumber filter. Symmetric DAC with anvil culet sizes of 300 µm and mineral oil was used as pressure transmitting medium. Pressure was also calibrated by the ruby luminescence method and silicon oil was used as the pressure transmitting medium (PTM).

### Theoretical Calculation

In the present first‐principles calculations, the Vienna Ab‐initio Simulation Package (VASP) was employed based on the density functional theory.^[^ ^60^, ^61^ ^]^ The exchange‐correlation functional was treated by the generalized gradient approximation (GGA) and parameterized by the Perdew, Burkey, and Ernzerhof functional.^[^ ^62^ ^]^ Projector‐augmented wave (PAW)^[^ ^63^ ^]^ approach was used to describe the core electrons and their effects on valence orbitals. To conduct the van der Waals correction, the zero‐damping DFT‐D3 functional was employed,^[64^ ^]^ and the spin‐orbit coupling (SOC) was taken into account in all the calculations. The plane‐wave kinetic‐energy cutoff was set to 500 eV, and the Brillouin zone was sampled with the special k‐mesh generated by the Monkhorst‐Pack scheme with a k‐point spacing of 2π × 0.02 Å^−1^. The convergence tolerance was 10^−6^ eV for total energy and all forces were converged to be < 0.003 eV Å^−1^. Tight‐binding models were constructed based on maximally localized Wannier functions (MLWFs) using WANNIER90 code.^[^ ^65^ ^]^ The topological electronic structures were studied by the WANNIERTOOLS package.^[^ ^66^ ^]^

## Conflict of Interest

The authors declare no conflict of interest.

## Supporting information

Supporting Information

## Data Availability

The data that support the findings of this study are available from the corresponding author upon reasonable request.
