# Supplementary material for: Pressure‐Induced Superconductivity and Topological Quantum Phase Transitions in the Topological Semimetal ZrTe2
Source: Adv Sci (Weinh). 2023 Nov 9;10(35):2301332. doi: 10.1002/advs.202301332 (PMC10724415; doi:10.1002/advs.202301332)
Supplement: Supplementary file 1 — Supporting Information [file ADVS-10-2301332-s001.pdf]

ADVANCED SCIENCE
Open Access

Supporting Information

for *Adv. Sci.*, DOI 10.1002/advs.202301332

Pressure-Induced Superconductivity and Topological Quantum Phase Transitions in the Topological Semimetal $ZrTe_2$

Shihao Zhu, Juefei Wu, Peng Zhu, Cuiying Pei, Qi Wang, Donghan Jia, Xinyu Wang, Yi Zhao, Lingling Gao, Changhua Li, Weizheng Cao, Mingxin Zhang, Lili Zhang, Mingtao Li, Huiyang Gou, Wenge Yang, Jian Sun, Yulin Chen, Zhiwei Wang, Yugui Yao and Yanpeng Qi**

Supporting Information

Pressure-induced Superconductivity and Topological Quantum Phase Transitions in the Topological Semimetal $ZrTe_2$

Shihao Zhu#, Juefei Wu#, Peng Zhu#, Cuiying Pei, Qi Wang, Yi Zhao, Lingling Gao, Changhua Li, Weizheng Cao, Mingxin Zhang, Lili Zhang, Mingtao Li, Wenge Yang, Jian Sun, Yulin Chen, Zhiwei Wang, Yugui Yao, Yanpeng Qi**

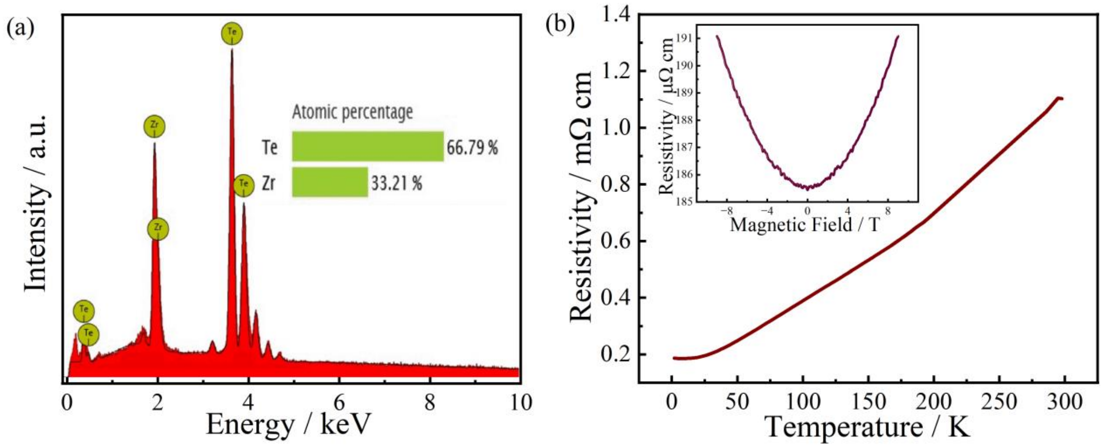

FIG. S1. (a) Energy-dispersive x-ray spectroscopy of $ZrTe_2$ (b) Temperature dependent resistivity of $ZrTe_2$ single crystal. Inset: magnetoresistivity of $ZrTe_2$ when ab-plane is perpendicular to magnetic field.

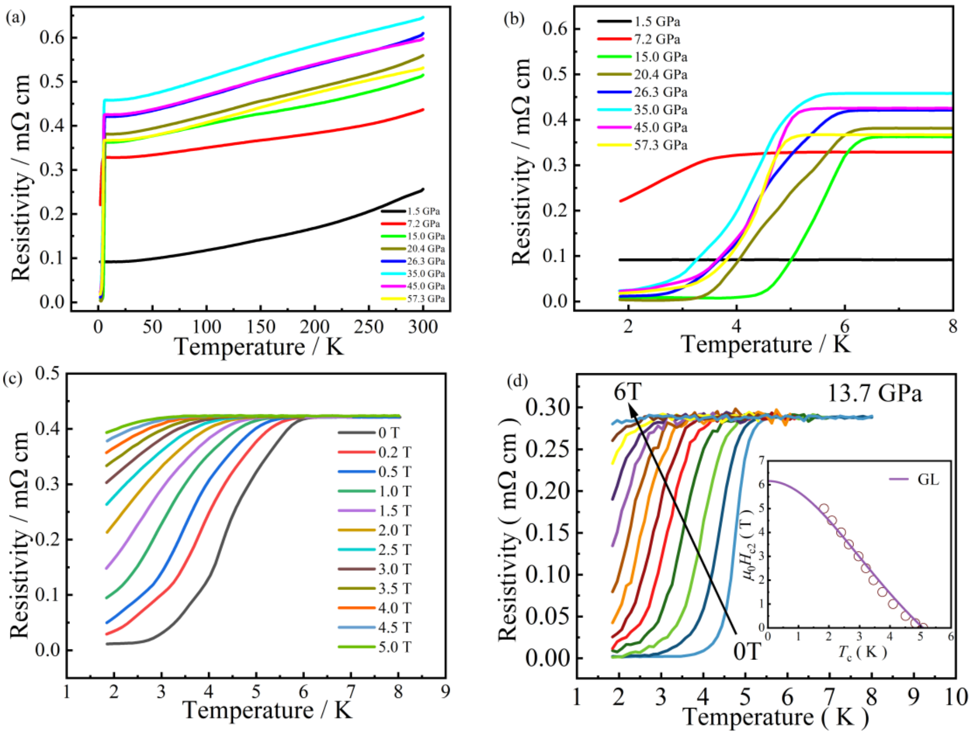

FIG. S2. (a) Electrical resistivity of $ZrTe_2$ as a function of temperature under pressures in run II. (b) Temperature-dependent resistivity of $ZrTe_2$ in the vicinity of the superconducting transition in run II. (c) Temperature dependence of resistivity under different magnetic fields for $ZrTe_2$ at 26.3 GPa in run II. (d) Temperature dependence of resistivity under different magnetic fields for $ZrTe_2$ at 13.7 GPa in run Ⅱ.

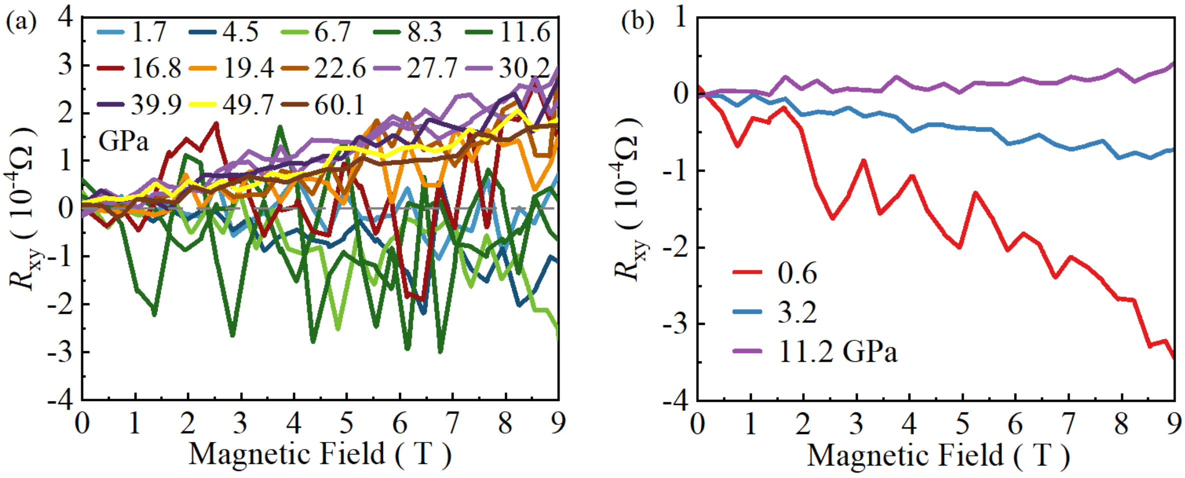

FIG. S3. (a) Hall resistance of $ZrTe_2$ as a function of magnetic field under various pressures at 10 K. (b) Hall resistance of $ZrTe_2$ as a function of magnetic field up to 11.2 GPa with sodium chloride as pressure transmitting medium at 10 K.

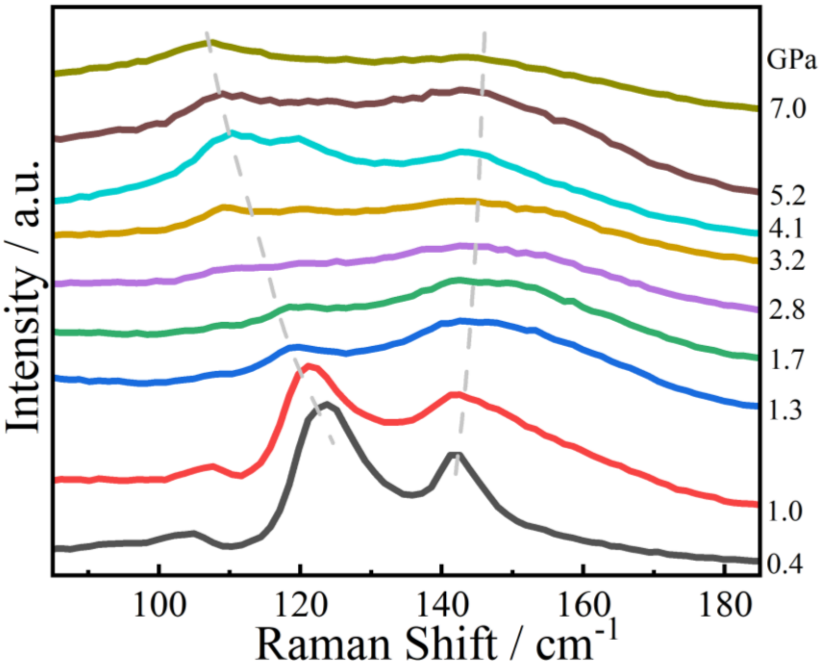

FIG. S4. Raman spectra at various pressures for $ZrTe_2$ at room temperature.

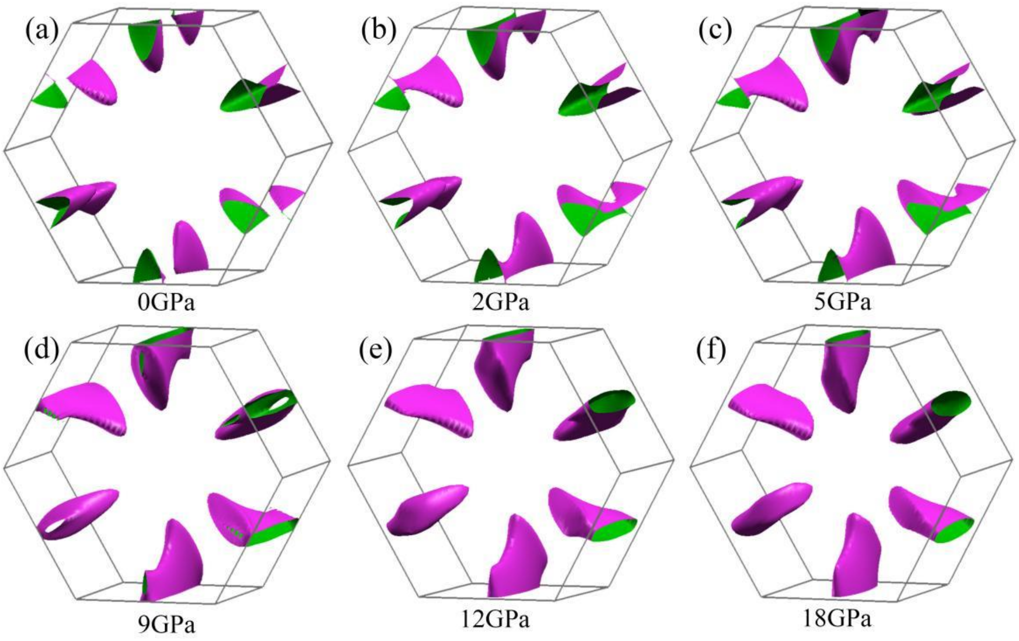

FIG. S5. The Fermi surface of the band I under different pressures.

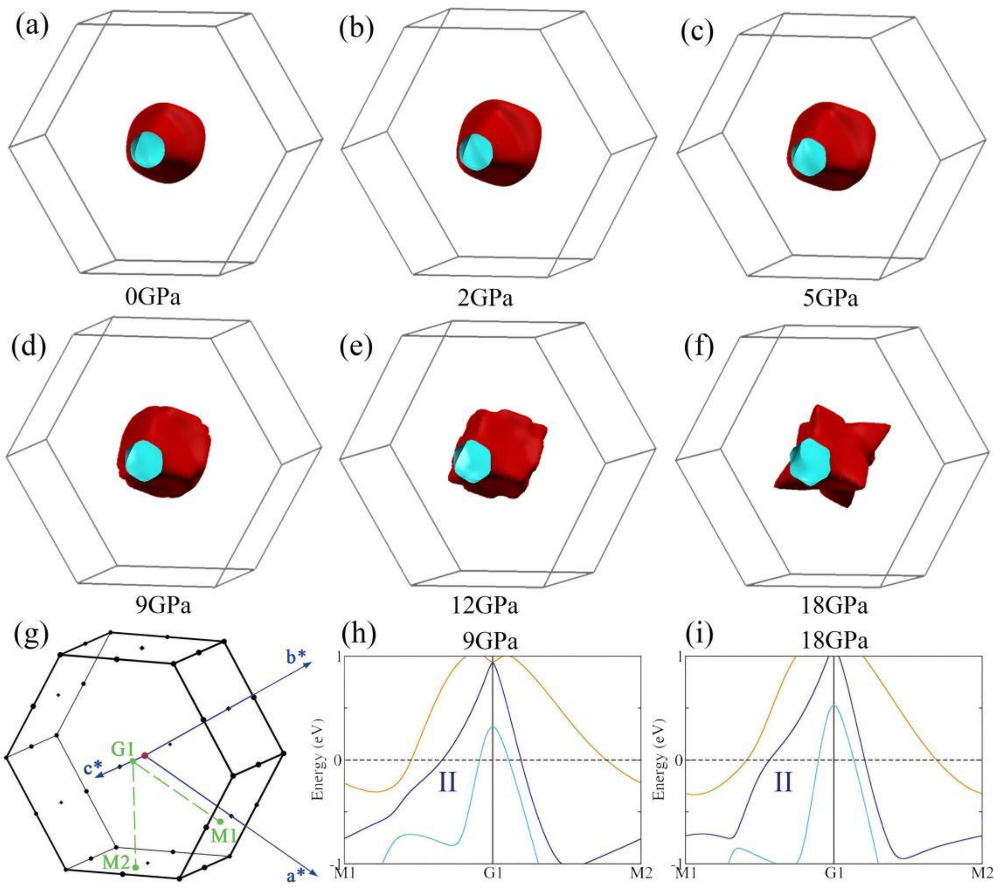

FIG. S6. (a)-(f) The Fermi surface of the band II under different pressures. (g) The Brillouin zone path on the $k_z = 0.25$ plane. The band structures at 9 GPa (h) and 18 GPa (i), the arrow points to a saddle point around the Fermi level at 18GPa (i).

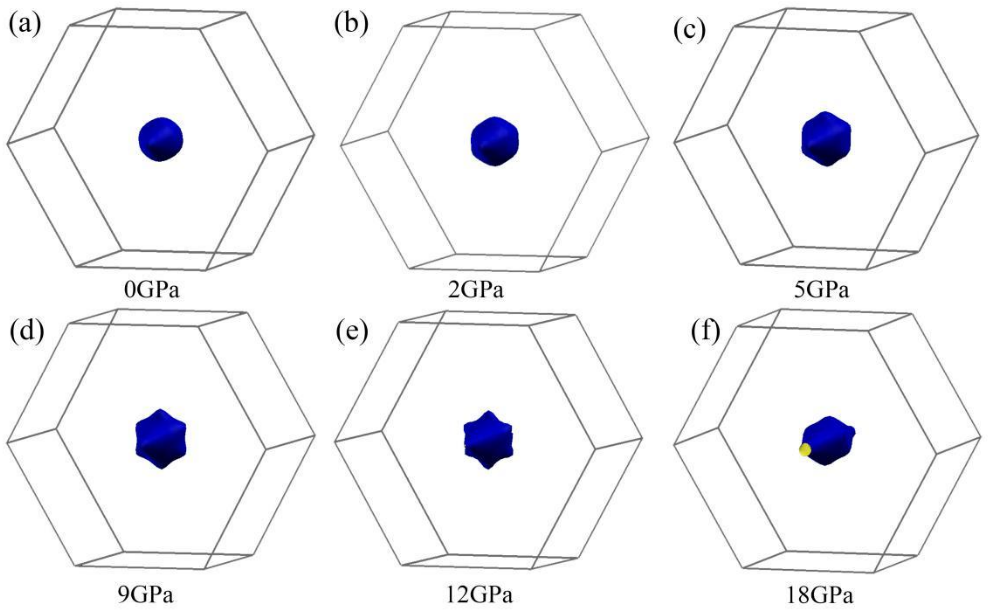

FIG. S7. The Fermi surface of the band III under different pressures.

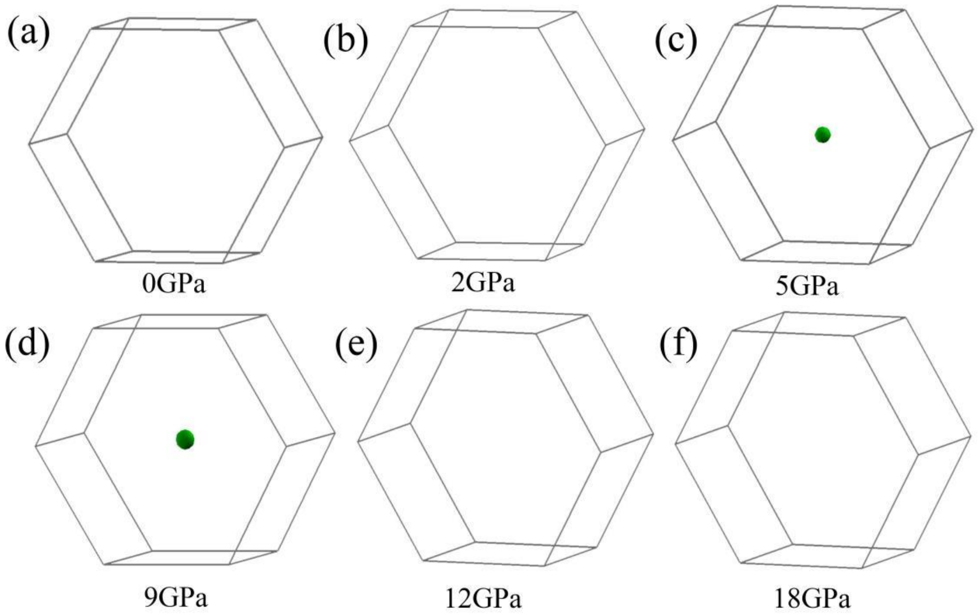

FIG. S8. The Fermi surface of the band IV under different pressures.

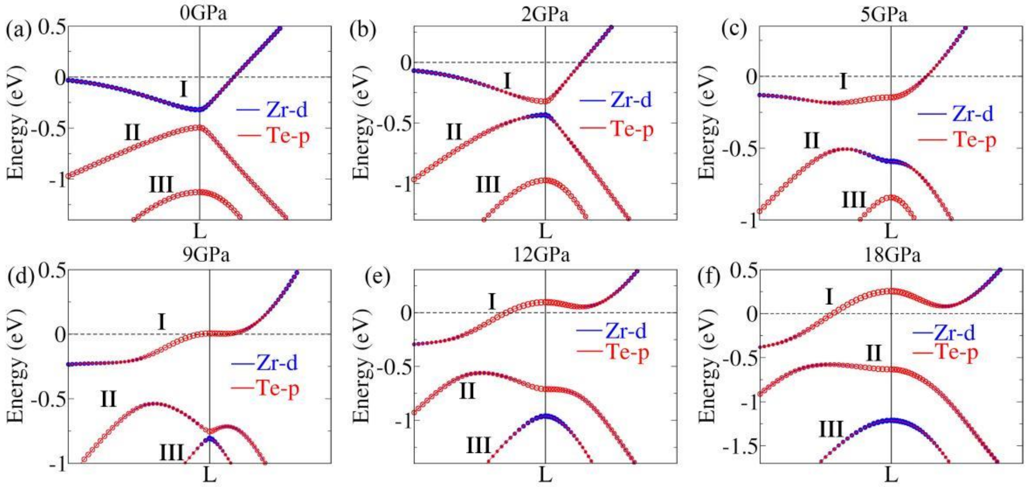

FIG. S9. The details of the band structures at *L* point, the blue points and the red points are the contribution from the *d* electrons of Zr atoms and the *p* electrons of Te atoms.

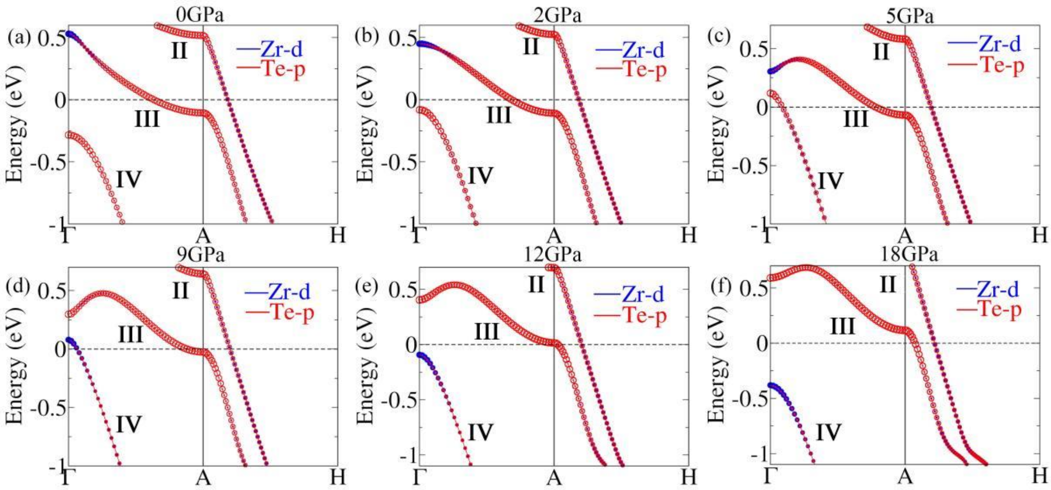

FIG. S10. The details of the band structures at *A* point, the blue points and the red points are the contribution from the *d* electrons of Zr atoms and the *p* electrons of Te atoms.

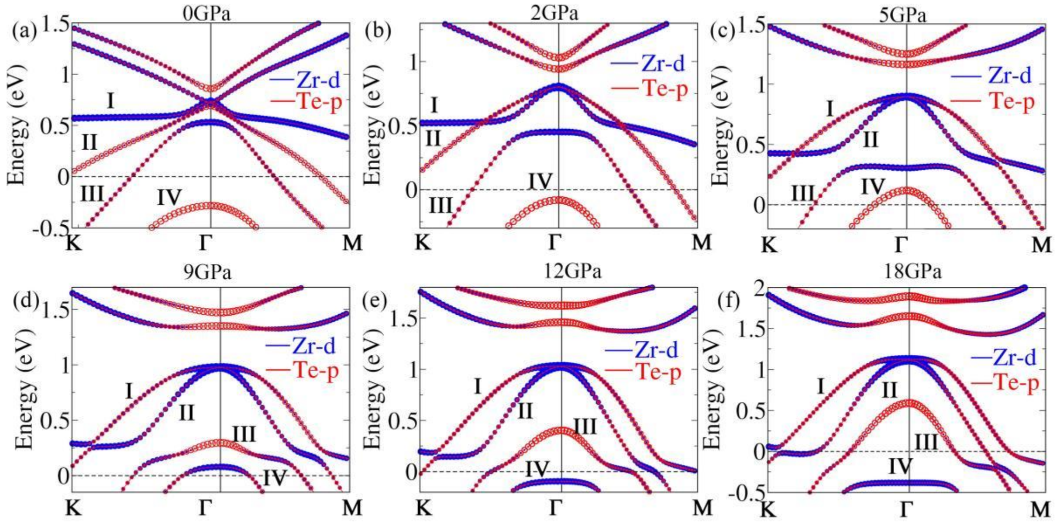

FIG. S11. The details of the band structures at *Γ* point, the blue points and the red points are the contribution from the *d* electrons of Zr atoms and the *p* electrons of Te atoms.

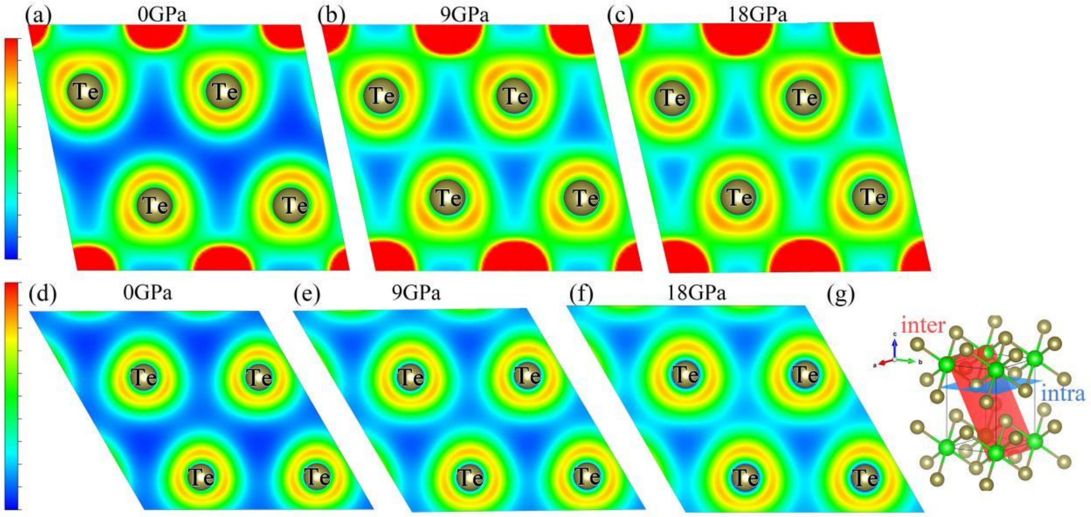

FIG. S12. The charge density between the inter-layer Te atoms (a)-(c) and the intra-layer Te atoms (d)-(f), the corresponding planes are in (g).

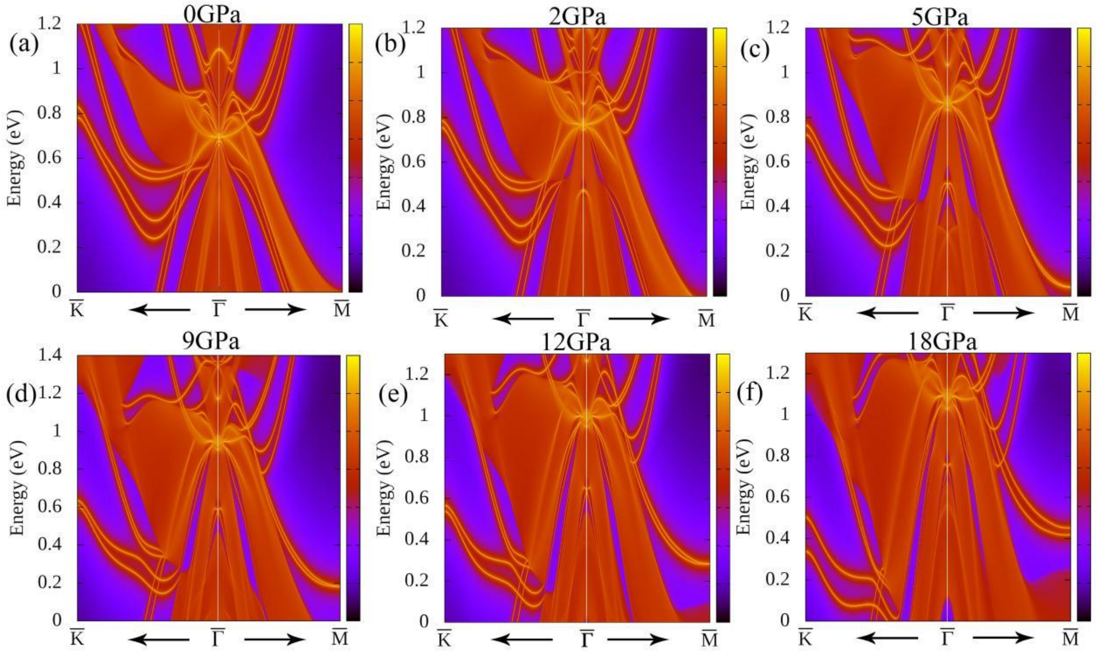

FIG. S13. The surface states on the (001) plane around $\bar{\Gamma}$ point at various pressures.

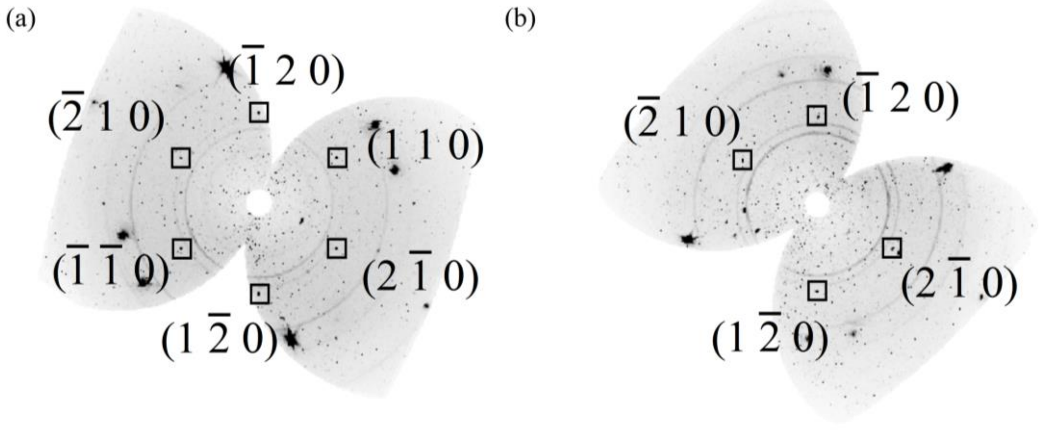

FIG. S14. single crystal XRD pattern of $ZrTe_2$ under (a) 5.8 GPa and (b) 14.3 GPa.

Table R1. Sample data and structure refinement for $ZrTe_2$ under 5.8 and 14.3 GPa.

Pressure of $ZrTe_2$	5.8 GPa		14.3 GPa	
Formula weight	364.42		364.42	
Temperature	296(2) K		296(2) K	
Wavelength	0.71073 Å		0.71073 Å	
Crystal system	Trigonal		Trigonal	
Space group	$P\bar{3}m1$		$P\bar{3}m1$	
	a = 3.9553 (11) Å	α = 90°	a = 3.926(2) Å	α = 90°
Unit cell dimensions	b = 3.9553 (11) Å	β = 90°	b = 3.926(2) Å	β = 90°
	c = 6.636 (7) Å	γ = 120°	c = 6.552(2) Å	γ = 120°
Volume	89.91 (11) $Å^3$		87.46(9) $Å^3$	
Z	1		1	
Density (calculated)	6.398 Mg/m^3		6.578 Mg/m^3	

Absorption coefficient	18.67 mm^{-1}	19.199 mm^{-1}
F(000)	144	144
Crystal size	0.035 x 0.032 x 0.027 mm^3	0.035 x 0.032 x 0.027 mm^3
θ range for data collection	6.0 to 22.2°	6.0 to 22.38°
Index ranges	-4<=h<=4, -3<=k<=3, -1<=l<=1	-4<=h<=4, -4<=k<=4, -1<=l<=1
Reflections collected	270	156
Independent reflections	18 [R(int) = 0.0570]	18 [R(int) = 0.1413]
Coverage of independent reflections	29.0 %	30.0 %
Refinement method	Full-matrix least-squares on F2	Full-matrix least-squares on F2
Data / restraints / parameters	18 / 6 / 6	18 / 12 / 6
Goodness-of-fit	1.588	1.020
Final R indices [>2σ(I)]	R1 = 0.0288, wR2 = 0.0559	R1 = 0.0781, wR2 = 0.1630
R indices [all data]	R1 = 0.0451, wR2 = 0.0608	R1 = 0.1196, wR2 = 0.1980
Largest diff. peak and hole	1.044 and -0.623 e.$Å^{-3}$	1.401 and -1.364 e.$Å^{-3}$
